# Postoperative Surgical Site Infections in the Department of General Surgery of a Tertiary Care Centre: A Descriptive Cross-sectional Study

**DOI:** 10.31729/jnma.7316

**Published:** 2022-05-31

**Authors:** Pradeep Ghimire, Binod Bade Shrestha, Om Bahadur Karki, Bishowdeep Timilsina, Ananda Neupane, Aprajita Bhandari

**Affiliations:** 1 Department of General Surgery, Manipal Teaching Hospital, Fulbari-11, Pokhara, Kaski, Nepal

**Keywords:** *cross-sectional studies*, *prevalence*, *surgical wound infection*

## Abstract

**Introduction:**

Surgical site infection is defined by the Centres for Disease Control and Prevention as a wound infection that occurs within 30 days of an operative procedure or within a year if an implant is left in place and the infection is thought to be secondary to surgery. It occupies 20% to 39% of all the infections acquired in hospitals. The aim of this study is to find out the prevalence of postoperative surgical site infections in the Department of General Surgery of a tertiary care centre.

**Methods:**

A descriptive cross-sectional study on a total of 384 post-operative patients of abdominal surgery was conducted in the Department of General Surgery of a tertiary care centre from August 1, 2020 to July 30, 2021 with ethical approval from the Institutional Review Committee (Reference number: 267). Convenience sampling was done. Post-operative patients fulfilling the inclusion and exclusion criteria were included in the study. Data were entered in Microsoft Excel and analyzed using the Statistical Package for the Social Sciences version 21.0. Point estimate was done at a 95% Confidence Interval along with frequency and percentages for binary data and mean with standard deviation for continuous data.

**Results:**

Among 384 patients, the prevalence of surgical site infection was found to be 65 (16.92%) (13.15-20.65 at a 95% Confidence Interval). The patients had a mean age of 42.06±21.92 years.

**Conclusions:**

The prevalence of surgical site infection was higher in our study in comparison to other similar studies conducted in similar settings.

## INTRODUCTION

Surgical Site Infection (SSI) is defined by the Centres for Disease Control and Prevention as a wound infection that occurs within 30 days of an operative procedure or within a year if an implant is left in place and the infection is thought to be secondary to surgery.^1^ SSI is a common nosocomial infection.^1,2^ It occupies 20% to 39% of all the infections acquired in hospitals.^3^ SSI may occur at any duration but mostly between the 5^th^ and 10^th^ days after surgery.^4^

SSIs are classified by The Centre for Disease Control (CDC) into (a) Superficial (b) Deep incisional (c) Organ/space.^5,6^ The common causative bacteria are *Staphylococcus aureus* (31.58%) followed by *Klebsiella pneumonia* (26.31%) and *Pseudomonas aeruginosa* (15.79%).^7^ Patients with SSI require more medical care and longer hospital stay.^8^

The aim of this study is to find out the prevalence of postoperative surgical site infections in the Department of General Surgery of a tertiary care centre in Nepal.

## METHODS

This was a descriptive cross-sectional study conducted in the Department of General Surgery, Manipal Teaching Hospital, Pokhara, Nepal from August 1, 2020 to July 30, 2021. Ethical approval was obtained from the Institutional Review Committee of Manipal College of Medical Sciences, Pokhara, before starting the study (Reference number: 267). Inclusion criteria were patients with abdominal surgeries (both elective and emergency operations) and patients providing written informed consent. Patients with abdominal surgeries other than general surgery (vascular, gynaecological, urosurgery) and patients with open laparotomy wounds were excluded. The convenience sampling method was used, and the sample size was calculated as follows:

n = (Z^2^ × p × q) / e^2^

  = (1.96^2^ × 0.13 × 0.87) / 0.04^2^

  = 287

Where,

n = minimum required sample size
Z = 1.96 at 95% Confidence Interval (CI)
p = prevalence of surgical site infections, 13.87%^9^
q = 1-p
e = margin of error, 4%

A minimum sample size of 287 was calculated. However, a sample size of 384 was taken for the study.

SSIs were diagnosed in the presence of pus or purulent discharge from the wound along with pain with any two cardinal signs of inflammation and clinically within 30 days of the operative procedure.^1^ A self-structured proforma was used as a data collection tool. Data were obtained from the hospital records department. Variables included in the study were patient demographic characteristics, possible preoperative risk factors (diabetes mellitus, immunosuppression and use of chemotherapy and steroid), smoking status, body mass index, and wound scoring with classification, and preoperative haemoglobin and albumin levels. Operative variables included operation performed, use of prophylactic antibiotic, wound contamination class, surgical approach (open or laparoscopic), the urgency of surgery and drain use. Data regarding admission to the intensive care unit, length of stay, postoperative complications and death were also recorded. Patients were followed up for 30 days in the ward, outpatient clinic or dressing clinic or through telephone interviews. Wound assessment was done with the Centres for Disease Control and Prevention and National Healthcare Safety Network definition of SSI.^1^

Surgical site infection was classified as superficial (involving the skin and subcutaneous tissue only), deep (involving deeper soft tissues such as fascia and muscle layers) or organ space (involving any part of the anatomy that was opened or manipulated during surgery).^5,6^ The infection was graded based on the South Hampton surgical infection grading and the Additional treatment, Serous discharge, Erythema, Purulent exudate, Separation of deep tissues, Isolation of bacteria and Stay as inpatient prolonged over fourteen days (ASEPSIS) wound grading score.

Data were entered in Microsoft Excel and analysed using the Statistical Package for the Social Sciences version 21.0. Point estimate was done at a 95% Confidence Interval along with frequency and percentage for binary data and mean with standard deviation for continuous data.

## RESULTS

**Figure 1:**
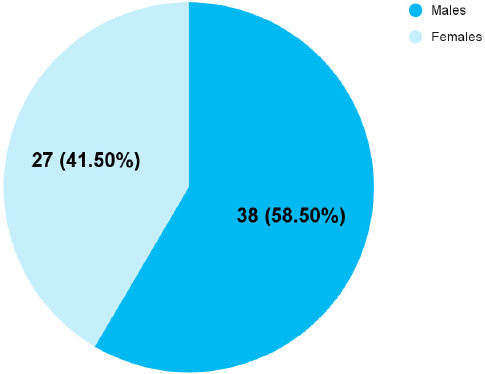
Gender-wise distribution of the patients with surgical site infection (n= 65).

Among 384 patients, the prevalence of surgical site infection was found to be 65 (16.92%) (13.15-20.65 at 95% Confidence Interval). The patients had a mean age of 42.06±21.92 years. In our study, 29 (44.62%) of SSI were seen in the adult age group (19-64 years), whereas 26 (40.00%) were seen in the old age group (>65 years) and 10 (15.38%) in the paediatric population (<18 years). SSI was found to be more common in males as compared to females (Figure 1).

Regarding the types of SSI, superficial SSI was present in 48 (73.84%), deep SSI was present in 13 (20.01%) and organ space SSI was present in 4 (6.15%) patients with SSI. Majority of the patients fell under Grade III of the South Hampton surgical infection grading system. Similarly, 28 (43.07%) patients comprised minor ASEPSIS wound grading scores (Table 1).

**Table 1:** Grading of the patients with SSI (n= 65).

South Hampton surgical infection grading	n (%)
Grade I	7 (10.78)
Grade II	14 (21.53)
Grade III	23 (35.38)
Grade IV	12 (18.46)
Grade V	9 (13.84)
**ASEPSIS wound grading score**	
Disturbance in healing (Scores: 11-20).	3 (4.61)
Minor (Scores: 21-30)	28 (43.07)
Moderate (Scores: 31-40)	18 (27.69)
Severe (Scores: ≥41)	16 (24.61)

Sixteen (24.61%) patients were obese whereas 49 (75.38%) were non-obese and 26 (40.00%) had existing comorbidities out of these 65 patients. Twenty-eight (43.07%) patients were major cases and 37 (56.92%) were intermediate cases. Fifty-four (83.08%) of SSIs were in open surgery and 11 (16.92%) of SSIs were in laparoscopic surgery. Forty (61.54%) cases were operated as emergency cases and 25 (38.46%) were operated as elective cases. In these patients, the total leucocyte count was found to be above the normal range (≥11000) in 48 (73.84%) patients. Similarly, the mean hospital stay was 8.07±4.90 days.

The patients were subjected to culture and sensitivity after which 58 (89.23%) patients showed growth of microorganisms whereas 7 (10.76%) patient culture didn't show any growth. The commonest organism isolated from the wound swab was *Staphylococcus aureus* in 29 (44.61%) patients followed by *Escherichia coli* in 18 (27.69%) patients and *Streptococcus epidermidis* in 9 (13.84%) patients (Table 2).

**Table 2:** Antibiotic sensitivity among patients with SSI (n = 58).

Antibiotics	n (%)
Inj. Meropenem	35 (60.34)
Inj. Tazobactam	31 (53.44)
Inj. Amoxiclav	25 (43.10)
Inj. Gentamycin	18 (31.03)
Inj. Tigecycline	10 (17.24)
Inj. Ciprofloxacin	7 (12.06)
Inj. Vancomycin	6 (10.34)
Inj. Cefotaxim	6 (10.34)
Inj. Erythromycin	5 (8.62)

## DISCUSSION

Surgical site infections are the second most common type of adverse event occurring in hospitalized patients following surgery and are one of the most common surgical complications.^10^ SSI surveillance is integral to hospital infection control and quality improvement programs, with feedback on SSI rates being an important component of SSI reduction strategies.^11^ The prevalence of SSI differs widely from hospital to hospital and from one geographic location to another.^12^ Surgical site infections are still one of the leading causes of morbidity and mortality among patients undergoing major surgery. SSIs remain a frequent postoperative complication; developing in 3% to 20% of surgical procedures.^13^

In our study, 65 patients developed SSI which was 16.92% of the enrolled patients. The rate of SSI in our centre is lower than in studies done in Pakistan where it was found to be 33.68%.^14^ It is still slightly higher than those reported in studies done in Saudi Arabia, 12%^15^ and 10.50%.^16^ In our study, the rate of SSI was more in adult groups and this result was obtained as more patients admitted and operated on were in adult age groups. A similar finding was present in two studies done in India.^17,18^

SSI was more common in male patients which could be due to multiple risk factors in males, such as cigarette smoking, alcohol, etc. It could also be due to improved immune function in females and differences in skin colonization between males and females.^19,20^ Obesity is also an important risk factor for the development of SSI. Obesity has been correlated with prolonged wound healing which is a known risk factor for SSIs.^21^ In our study, among patients with SSIs, 16 (24.61%) were obese whereas 49 (75.38%) were non-obese. This variation could be due to more number of patients admitted and operated on were of the non-obese group.

Our study also concluded that SSI was more common in emergency surgery which could be due to the contaminated and dirty wounds and higher bacterial load in emergency cases as compared with a clean and clean-contaminated wound in elective cases. Emergency surgeries may have to be performed in less than ideal conditions and without usual preoperative workup which might contribute to higher SSI in these cases. Also, these surgeries are more likely to be associated with cross-contamination and poor general health of patients. A similar finding was found to be present in the study done in Africa.^21^

Patients with comorbidities had a greater percentage of SSIs as compared to patients without comorbidities. In our study, among patients with SSIs, 26 (40.00%) had existing comorbidities. A study conducted at a teaching hospital in a tertiary care centre in Eastern India showed an association of co-existing comorbidities in the development of SSIs.^22^ Among the type of surgery, in major cases usually there is increased operative time, more tissue handling, and increased blood loss than in intermediate cases which is prone to developing SSI. In our study, among patients with SSIs, 28 (43.07%) were major cases 37 (56.92%) were intermediate cases. This variation might have been present due to more number of patients admitted and operated were in Intermediate surgery groups.

Among laparoscopic and open surgery, open surgery had greater chances of developing SSI. A similar finding has been studied.^23^ In laparoscopic surgery, a minimal impact on the immune system, minimal exposure to the external environment, carbon dioxide pneumoperitoneum, better visualisation of tissues for dissection and haemostasis are common aspects that may reduce the occurrence of SSI.^24^ We found that the development of SSI was more in patients with prolonged lengths of hospital stay which can be supported by another similar study.^25^ It could be because of a greater incidence of contamination of the patient during the hospitalization period which facilitates the development of infectious processes.

The microbiological profile of the infected wound showed growth of *Staphylococcus aureus* to be the most common which can be supported by another study as well and this could be because the majority of the cases were of superficial type.^26^ Also, it is noteworthy that *Escherichia coli* was the second most prevalent microorganism in our study. In our study, Injection Meropenem was found to be sensitive in most the patients with SSIs which could be because of overuse of the antibiotics like 3^rd^ generation cephalosporins and quinolones in our region and setting which might be the cause for the development of resistance in such group of drugs and the development of sensitivity to broad-spectrum antibiotics.

One of the major limitations of this study was that it was limited to a single centre, so the results might not apply to other patient populations. Hence, a multicentre study would be more fruitful invalidating the results of this study. This was a descriptive crosssectional study, so a comparison between groups could not be done and association with risk factors could not be established.

## CONCLUSIONS

The prevalence of surgical site infection was higher in our study in comparison to other similar studies conducted in similar settings. Among the SSI cultures, the most prevalent microorganism was *Staphylococcus aureus* followed by *Escherichia coli* and the most sensitive antibiotic was found to be Meropenem. Studies regarding the risk of developing SSI in patients undergoing general surgery are encouraged for enforcement of preventive measures to reduce infection rates. Also, new studies using different methodologies should be carried out in different circumstances that would add knowledge about SSI in general surgeries.
